# Impact of health-promoting text messages on cardiovascular risk: findings from a randomized controlled trial in primary care

**DOI:** 10.3389/fmed.2026.1807519

**Published:** 2026-05-21

**Authors:** Amanda Björk Javanshiri, Beata Borgström Bolmsjö, Moa Wolff, Hanna Glock, Veronica Milos Nymberg, Peter Nymberg, Sara Modig, Susanna Calling

**Affiliations:** 1 Center for Primary Health Care Research, Department of Clinical Sciences, Lund University, Malmö, Sweden; 2 University Clinic Primary Care Skåne, Skåne University Hospital, Region Skåne, Sweden

**Keywords:** cardiovascular diseases, healthy lifestyle, primary health care, primary prevention, randomized controlled trial, risk factors, text messaging

## Abstract

**Introduction:**

Cardiovascular disease (CVD) prevention is a major public health priority. Text message interventions promoting a healthy lifestyle can contribute to improved blood pressure in patients with hypertension, however the effect on other metabolic outcomes and broader CVD risk factor control are less studied. We aimed to investigate the effect of health-promoting text messages on metabolic- and multiple CVD risk factors among Swedish primary care patients with hypertension.

**Methods:**

This was a multi-center randomized controlled trial including a total of 401 participants. Four health-promoting text messages per week were sent to the intervention group (*n* = 193) for 6 months, with the control group (*n* = 208) receiving standard care. Data were analyzed according to the intention-to-treat principle. We investigated the intervention effect on metabolic risk factors, change in number of CVD risk factors, and improvements in CVD risk factor control at follow-up compared to baseline.

**Results:**

There was no difference between the intervention and the control group in improved CVD risk profiles, although both groups had decreased their number of CVD risk factors at follow-up. However, in subgroup analyses both sedentary participants and those with poor self-rated health demonstrated significant improvements in CVD risk factor control between baseline and follow-up in the intervention compared with the control group (Z = -2.551, *p* = 0.01; *Z* = −2.043, *p* = 0.041). Furthermore, the intervention group had a modest reduction in HbA1c (−0.61 mmol/L, *p* = 0.04), with limited clinical impact.

**Discussion:**

Health-promoting text messages did not reduce the number of CVD risk factors or improve the overall risk profile in the intervention group compared to control but showed a small effect among participants with sedentary lifestyle or poor self-rated health. Its overall effect on metabolic risk including HbA1c was limited. Still, modest effects on CVD risk factors may have important public health implications, especially in targeted groups.

**Clinical trial registration:**

ClinicalTrials.gov, identifier NCT04407962.

## 1 Introduction

Cardiovascular disease (CVD) remains the leading cause of death worldwide, posing a major public health challenge (1). Key risk factors for CVD include metabolic risk factors such as hypertension, dyslipidemia, hyperglycemia, obesity, in addition to unhealthy lifestyle habits (2). Notably, modifiable risk factors such as unhealthy diet, physical inactivity, and tobacco use contribute to 80% of CVD cases (3). Adopting healthy lifestyle habits can significantly lower this risk, as is also emphasized by European guidelines (4–6). However, it is well known that many patients struggle to adhere to lifestyle recommendations and that implementation of primary prevention strategies remain suboptimal among individuals with increased CVD risk (7–9). In Sweden, primary care treats a variety of patients with increased risk of CVD and has a central role in promoting healthy lifestyle changes as part of both primary and secondary prevention. With limited resources in primary care and mixed evidence for individual lifestyle counselling, digital interventions such as text messages to specific risk groups have emerged a simple, low-cost, and scalable tool to reach a broad primary care population (10–12). Earlier research has shown that text messaging interventions modestly affect CVD risk factors, especially blood pressure (11, 13–15). However, other risk factors such as body mass index (BMI), lipids, and HbA1c, have been less studied. Furthermore, it has been difficult to quantify the extent to which mobile phone interventions improve CVD risk factors or prevent disease outcomes, likely due to the heterogeneity among studies (16). It is reasonable to believe that simultaneous but small impacts on multiple CVD risk factors could produce an additive effect that might improve outcome. Nevertheless, there is a lack of studies in primary care investigating the effectiveness of text messaging in assisting lifestyle improvements aimed at reducing multiple CVD risk factors at the same time. We have previously analyzed whether health-promoting text messages could affect blood pressure and lifestyle habits in a Swedish primary care population, and found small effects on diastolic blood pressure in certain groups (sedentary individuals and those with poor self-rated health) as well as physical activity and alcohol use (14, 17). However, the effects on metabolic risk other than blood pressure, and on multiple CVD risk factors in combination have not been analyzed. We aimed to investigate the effect of health-promoting text messages on metabolic- and composite CVD risk factors among primary care patients with hypertension, in a randomized controlled study.

## 2 Materials and methods

### 2.1 Study design and setting

**Figure 1:**
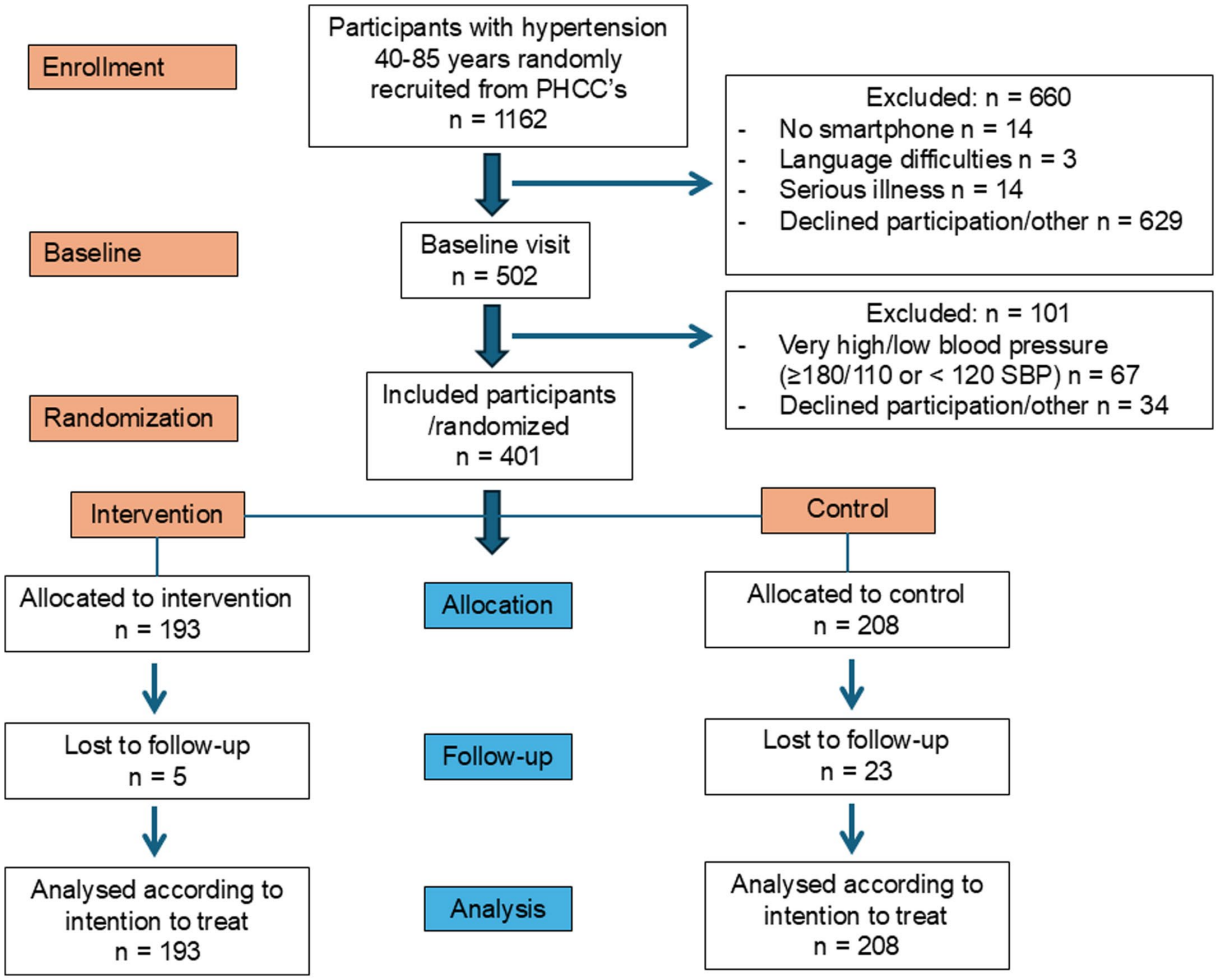
CONSORT flow diagram showing participant progress through the trial.

This study examines the secondary outcomes of the PUSHME (Primary care USage of Health promoting MEssages) multi-center randomized controlled trial (RCT), which included a population of 401 patients with hypertension (International Classification of Diseases code I10.9). The primary endpoint was difference in blood pressure, and those results have already been published (14). Participants were randomly recruited from 10 primary healthcare centers in four southern regions of Sweden between the 1st of September 2020 and December 2022, with the last follow-up visit on the 15th of June 2023. The study included one baseline visit and a single follow-up conducted at 6 months. No additional follow-up visits were scheduled. The 6-month interval was chosen to allow sufficient time for measurable changes in the study outcomes while still minimizing participant burden and loss to follow-up. The inclusion criteria were a diagnosis of hypertension, owning a smartphone, and being between 40 and 85 years old. Age range was based on the assumption that essential hypertension is uncommon in individuals under 40 years of age and that multi-morbidity and mortality increases substantially in those older than 85 years. Also, the intervention consisted of lifestyle advice that could be potentially harmful for vulnerable individuals. Participants with short life expectancy (below one year), language difficulties, cognitive impairment, or very high/low blood pressure (≥180/110 or < 120 systolic blood pressure at baseline) were excluded. The study procedures and flow diagram of included participants have previously been described in detail (14). Written informed consent was obtained prior to inclusion. The study adhered to the CONSORT (Consolidated Standard of Reporting Trials) reporting guidelines and Figure 1 illustrates a flow diagram of the study population (18).

### 2.2 Randomization

A 1:1 computer-generated predefined block randomization was used to assign participants to receive text messages in addition to standard care or standard care only. Standard care entailed that the control group continued with their prescribed antihypertensive medication and participated in a baseline visit (where no lifestyle advice was given), aside from their annual check-up with their general practitioner if it happened to occur during the study period. The patient’s general practitioner and study nurse were blinded to the group allocation.

### 2.3 Intervention

Four lifestyle-promoting text messages per week were sent to the intervention group, sometime between 9 a.m. and 7 p.m., for six months. These contained information, advice, and educational links about different lifestyle habits, covering the following domains:

1. Physical activity,
2. Diet,
3. General cardiovascular health tips including alcohol consumption, and
4. Tobacco use (for smokers only).

All messages were based on the Swedish national lifestyle recommendations, designed according to motivational interviewing, edited by experts and further improved after feedback from the participants in a pilot study (19). Participants who smoked received one text message per week from each lifestyle domain. For non-smokers, the tobacco message was replaced by an additional message from domain three. The messages were also personalized by staring with participant’s first name (14). Example of text message content from group three: “cardiovascular disease is the most common cause of death in Sweden and many other countries, and high blood pressure is an important risk factor. You can reduce your risk by improving your lifestyle habits!*”*

### 2.4 Sample size

The sample size for the main trial was determined based on the primary outcome blood pressure effect estimates and variability observed in the preceding pilot study (19). Power calculation was based on assumed 80% statistical power with a two-sided test, a 5% significance level, an expected between-group difference of 4 mm Hg, a standard deviation of 13 mm Hg, and a dropout rate of 10% (14).

### 2.5 Outcomes

The present study first assessed the effect of the intervention on metabolic risk factors that had not been previously analyzed in the RCT material; non-HDL cholesterol, total cholesterol, HbA1c, BMI, weight and waist circumference.

Then we analyzed the effect of the intervention on a combination of CVD risk factors. The risk factors were defined based on previous research and established guidelines as: high blood pressure (≥130/80 mmHg), hyperlipidemia (non-HDL ≥ 3.4 mmol/L), overweight (BMI > 25 kg/m^2^), and physical inactivity (<150 activity minutes per week) (6, 20–22). Here, the outcome was number of CVD risk factors ranging from 0-4 depending on how many of the above risk factors were present.

Finally, we analyzed the effect of the intervention on CVD risk factor improvement. Improvement with regards to the different risk factors was defined as a 4 mmHg decrease in systolic and/or diastolic blood pressure, a 10% decrease in non-HDL cholesterol, a 2.5% weight reduction, and an increased physical activity level of 1 step on a previously defined 15-step scale (23, 24). This resulted in an ordinal variable of the number of improved CVD risk factors (0–5). Levels of improvement were prespecified based on prior evidence that small reductions in blood pressure, modest weight loss, and proportional decreases in atherogenic lipoproteins translate into clinically meaningful cardiovascular risk reductions, and that increases in physical activity are associated with lower cardiovascular risk (19, 25–26).

### 2.6 Measurements

Blood pressure, weight, and height were measured by a study nurse at the baseline and 6-month follow-up visit. Blood pressure was measured according to established guidelines in European Society of Hypertension, i.e., on the right arm of the seated participant after 5–10 min of rest by an electronic monitor (Boso Medicus) on two occasions, and the mean value was used. In case of a large discrepancy (> 5 mmHg) between the measurements, a third measurement was taken and the mean BP calculated (27). BMI was divided into normal weight (<25 kg/m^2^), overweight (25.00–29.9 kg/m^2^), and obesity (≥30 kg/m^2^). Blood tests for analysis of HbA1c, total cholesterol, LDL- and HDL cholesterol were also collected. Non-HDL cholesterol was calculated by subtracting HDL cholesterol from total cholesterol. Physical activity was measured by a self-reported questionnaire at baseline and follow-up with two validated questions from the Swedish National Board of Health and Welfare, covering moderate intensity exercise ranging from 0 to > 300 min per week and vigorous-intensity exercise with response options ranging from 0 to >120 min per week. To calculate weekly physical activity, we used the formula (2*vigorous + moderate activity) with midpoint values and a cut-off for physical inactivity at < 150 min/week according to international recommendations (23, 24, 28).

### 2.7 Statistical analysis

The data were analyzed according to the intention-to-treat principle, based on a predefined statistical analysis plan, using IBM SPSS version 31.0.0.0. All outcomes reported in the present analyses were defined as secondary outcomes and therefore not included in the primary power calculation. Given the exploratory nature of these secondary outcomes, no formal adjustment for multiple comparisons was applied. We analyzed the change in metabolic risk factors, total number of CVD risk factors, and improvement in CVD risk factors between the intervention and control groups at follow-up compared to baseline. After checking for assumptions, individual samples’ t-tests were used for continuous variables (metabolic risk factors), creating delta variables of change between follow-up and baseline values. To analyze changes in CVD risk factors, we dichotomized the variables according to the above threshold values indicating elevated CVD risk. Then we created a composite ordinal variable of number of CVD risk factors. Ordinal logistic regression with adjustment for baseline values was used to analyze the change in the number of CVD risk factors. The Wilcoxon Singed-Rank test was used to compare median within the groups. For the variable improvement in CVD risk factors, binary logistic regression adjusted for baseline values was used as well as a non-parametric Mann–Whitney U test. Then we conducted subgroup analyses, according to prespecified subgroups (participants with sedentary lifestyle, poor self-rated health, diabetes, previous CVD, and BMI > 30), since previous studies on the same data indicated better effects of the intervention in these subgroups, which might be clinically relevant (14, 19). Missing data for patients lost to follow-up were imputed with the last observation carried forward. As a sensitivity analysis, a complete case analysis was also performed.

## 3 Results

Among 1,162 invited patients, 401patients consented and met the inclusion criteria. Participants consisted of both female and male individuals. They were randomized to either the intervention group (*n* = 193) or the control group (*n* = 208). For the complete flow chart of the study population and recruitment, we refer to a previously published study (14). At follow-up, 188 participants remained in the intervention group and 185 in the control group, with a total dropout rate of 7.2% (29/401). Baseline characteristics are presented in Table 1 and showed no significant differences between the groups.

**Table 1:** Baseline characteristics of the study population, by treatment group and total.

Characteristics	Total *n* = 401 (%)	Intervention *n* = 193 (%)	Control *n* = 208 (%)
Age, years	68.9 ± 9.4	68.2 ± 8.9	69.0 ± 9.8
Women	191 (47.6)	88 (45.6)	103 (49.5)
Men	210 (52.4)	105 (54.4)	105 (50.5)
SBP, mmHg	140.5 ± 13.0	140.4 ± 13.0	140.5 ± 13.1
DBP, mmHg	84.1 ± 10.0	83.5 ± 9.4	84.6 ± 10.7
BMI kg/m^2^	28.6 ± 5.0^1^	28.4 ± 4.8^1^	28.7 ± 5.1
BMI < 25	104 (26.0)	52 (27.1)	52 (25.0)
BMI 25–29.9	168 (42.0)	82 (42.7)	86 (41.3)
BMI ≥ 30	128 (32.0)	58 (30.2)	70 (33.7)
HbA1c (mmol/L)	39.6 ± 6.7	40.1 ± 6.8	39.3 ± 6.6
non-HDL (mmol/L)	3.4 ± 1.1^1^	3.4 ± 1.2	3.5 ± 1.1^1^
Diabetes	65 (16.2)	36 (18.7)	29 (13.9)
Sedentary < 150 physical activity minutes/week	128 (31.9)	62 (32.1)	66 (31.7)
Physical activity minutes per week^2^	240.5 ± 152.2	243.7 ± 151.3	237.3 ± 153.3
Previous CVD event^3^	58 (14.5)	28 (14.5)	30 (14.4)
Family history of hypertension	264 (65.8)	128 (66.3)	136 (65.4)
Alcohol > 4 standard drinks/week^4^	104 (25.9)	47 (24.4)	57 (27.4)
Current smoker	17 (4.2)	10 (5.2)	7 (3.4)
Upper secondary/higher education	264 (65.8)	139 (72.0)	153 (73.6)

Continuous variables are presented as means and standard deviations, categorical variables are presented as frequencies and percentages.

mmHg, millimeters of mercury; kg, kilogram; m, meter; mmol, millimole; L, liter; CVD, cardiovascular disease.

^1^One patient’s data missing.

^2^Calculated as (2*vigorous + moderate activity) from questionnaire, answer options with ranges of minutes/week.

^3^Previous myocardial infarction, stroke, angina pectoris, aortic aneurysm, operation CABG or angioplasty.

^4^Questionaire answer options of standard drinks/week: <1, 1–4, 5–9, 10–14, 15–19, >19.

### 3.1 Metabolic risk factors

Change in metabolic risk factors between baseline and follow-up are presented in Table 2. There were no differences in mean change in non-HDL-cholesterol, total-cholesterol, BMI, weight, or waist circumference between the intervention and the control-group at follow-up. However, there was a statistically significant decrease in HbA1c in the intervention group compared to the control group (−0.61 mmol/L, *p* = 0.04).

**Table 2:** Mean change in metabolic risk factors for intervention and control group from baseline to follow-up.

Metabolic risk factors	Intervention *n* = 193	Control *n* = 208	*p*-value*
nonHDL cholesterol (mmol/L)	−0.16 ± 0.82	−0.11 ± 0.58^1^	0.41
Total cholesterol (mmol/L)	−0.19 ± 0.75	−0.11 ± 0.61	0.28
HbA1c (mmol/L)	−0.34 ± 2.96	0.27 ± 3.12	**0.04**
BMI (kg/m2)	−0.60 ± 0.96^1^	−0.09 ± 0.78	0.44
Weight (kg)	−0.48 ± 2.87	−0.28 ± 2.3	0.45
Waist circumference (cm)	−0.39 ± 3.76	−0.87 ± 4.59	0.26

*Difference between mean change in intervention and mean change in control group, analyzed with independent samples T-test, presented as mean ±SD, standard deviation.

^1^One patients’ data missing at baseline.Bold values indicate statistically significant results (*p* < 0.05).

### 3.2 Number of CVD risk factors

**Figure 2:**
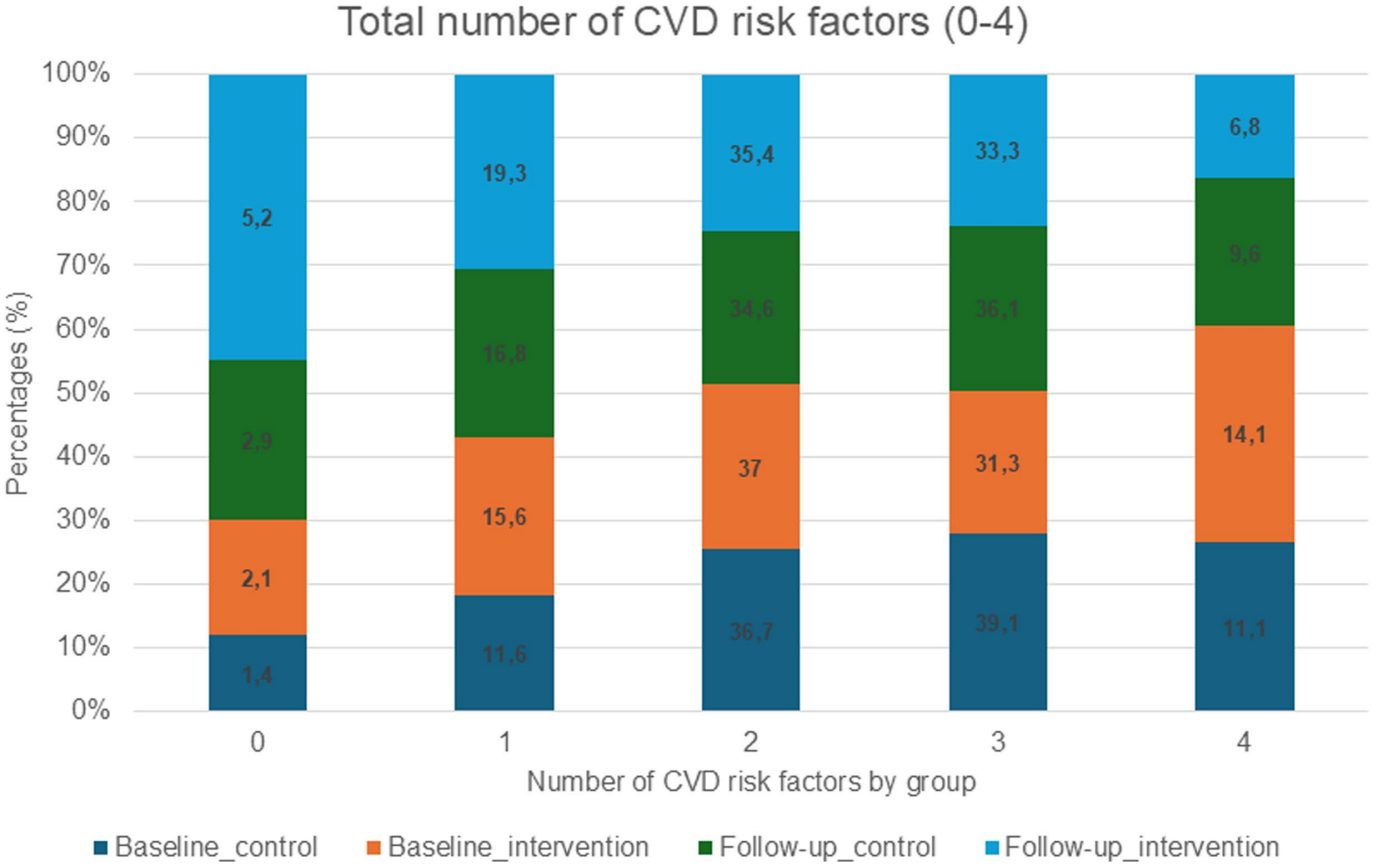
Proportions of CVD risk factors in the intervention and the control group (*N* = 401).

Participants in the intervention group had a lower median number of CVD risk factors (BP ≥ 130/80 mmHg, non-HDL ≥ 3.4 mmol/L, BMI > 25 kg/m^2^, and physical inactivity <150 activity minutes per week) at baseline [2 (3–2)] compared to the control group [3 (3–2)], however this difference was not statistically significant (Mann–Whitney U = 18959.500, *p* = 0.404, *r* = 0.04). Proportions of CVD risk factors in the intervention and control groups at baseline and follow-up are presented in Figure 2.

An ordinal logistic regression was performed to assess whether follow-up risk category (0–4) differed between the intervention and control groups, adjusting for baseline. The model showed that baseline risk was a significant predictor of follow-up risk category (*B* = 1.97, SE = 0.14, *p* < 0.001), with higher baseline scores associated with higher odds of being in a higher risk category at follow-up. However, there was no significant difference between the intervention and control groups in follow-up risk category (*B* = 0.28, SE = 0.19, *p* = 1.51). Although no significant difference was observed between the groups, both groups showed a comparable significant decrease when performing a Wilcoxon Signed-Rank test (*Z* = −2.656, *p* = 0. 008, *r* = 0.13; *Z* = −3.705, *p* = <0.001, *r* = 0.18). No significant differences were found when performing the subgroup analyses as stated in methods.

### 3.3 Improvement in CVD risk factors

Subsequently, we analyzed the numbers and proportions of improvement in CVD risk factors at follow-up compared to baseline, see Table 3. The intervention was associated with higher odds of increased physical activity (OR = 2.0, 95% CI 1.313–3.118), compared with the control group, which also has been previously reported (17). In general, participants in both arms made several improvements in their CVD risk factors during the study period by decreasing their blood pressure, weight, non-HDL cholesterol levels or increasing their physical activity, but there was no median difference in total number of improved risk factors between the groups.

**Table 3:** Numbers and proportions of improvement in CVD risk factors at follow-up compared to baseline.

Improved CVD risk factor	Intervention *n* = 193 (%)	Control *n* = 208 (%)	*p*-value	OR
≥ 4 mmHg decrease in SBP	90 (46.6)	99 (47.6)	0.863^1^	0.965
≥ 4 mmHg decrease in DBP	81 (42.0)	80 (38.5)	0.298^1^	1.246
≥ 2.5% weight reduction	42 (21.8)	31 (14.9)	0.075^1^	1.595
≥ 10% decrease in non-HDL cholesterol	57 (29.5)	57 (27.5)^3^	0.587^1^	1.129
Increased physical activity level^*^	91 (47.2)	70 (33.7)	**0.001** ^1^	2.023
Median improvement in CVD risk factors^**^	2.0	2.0	0.057^2^	

^*^An increased physical activity level of one step on a previously defined 15-step scale.

^**^Score 0–5 where each variable specified in the table renders 1 point if improved.

^1^Logistic regression with adjustment to baseline.

^2^Mann–Whitney U test comparing overall improvement scores between groups.

^3^One patient’s data missing at baseline.Bold values indicate statistically significant results (*p* < 0.05).

However, in the subgroup analyses, sedentary participants and those with poor self-rated health (SRH) showed a significant improvement in CVD risk factors at follow-up in the intervention group compared with the control group (*Z* = −2.358, *p* = 0.018, *r* = 0.12 and *Z* = −2.043, *p* = 0.041, *r* = 0.10), indicating a small effect size according to conventional benchmarks (with *r* < 0.1 considered trivial and *r* = 0.1–0.3 considered small; Cohen, 1988) (29), as shown in Table 4.

**Table 4:** Subgroup analyses comparing improvement in CVD risk factors among sedentary individuals and participants with poor-SRH.

Subgroup	Group	*n*	Median (IQR)	U	*Z*-value	*p*-value^*^	*r*
Active individuals^1^	Intervention	131	2.0 (2–1)	8727.500	−0.807	0.420	0.04
	Control	141	1.0 (3–0)				
Sedentary individuals	Intervention	62	2.0 (3–1)	1563.500	−2.358	**0.018**	0.12
	Control	66	2.0 (3–1)				
Good SRH^2^	Intervention	136	2.0 (3–1)	9147.500	−0.880	0.379	0.04
	Control	143	2.0 (3–1)				
Poor SRH	Intervention	64	2.0 (3–1)	1440.500	−2.043	**0.041**	0.10
	Control	57	1.0 (2.75–0)				

^*^Comparing subgroups of the intervention and control groups at follow-up.

^1^Defined as > 150 activity minutes per week.

^2^Defined as 1–2 measured on a Likert scale ranging from 1 to 5 (1 = very good, 5 = very poor).

U, Mann–Whitney U; IQR, interquartile range; *r*, effect size.Bold values indicate statistically significant results (*p* < 0.05).

## 4 Discussion

This study investigated the effects of health-promoting text messages on metabolic- and composite CVD risk factors among patients with hypertension. Overall, the study population showed a reduction in CVD risk factors during the study period, but there was no difference between the groups. The intervention significantly improved CVD risk profiles among participants who were sedentary or reported poor self-rated health at baseline. Furthermore, the intervention group had a modest reduction in HbA1c, that might be associated with increased physical activity.

In the previously published TEXT ME RCT from 2015, participants with established coronary heart disease (secondary prevention) who received weekly text messages were nearly twice as likely to achieve multiple CVD risk factor control compared to those receiving standard care (10). However, the subsequent TextMe2 RCT, which focused on primary prevention in individuals without known coronary heart disease, found that while more participants in the intervention group reduced the overall number of uncontrolled CVD risk factors and were less likely to be physically inactive compared to controls, there was no significant difference in the proportion of patients with three or more uncontrolled risk factors, nor in other single risk factor changes at 6-month follow-up (11). Notably, the TextMe2 population consisted of patients referred to a cardiology clinic due to chest pain, thus being a potentially more motivated cohort than the primary care population with hypertension in our study. Nonetheless, both populations consisted of participants with low to moderate CVD risk, and our study shows similar results as TextMe2. On the other hand, another Australian study, the larger SupportMe RCT from 2019, investigating the effect of a similar text messaging program in patients with type 2 diabetes (T2DM) and/or chronic heart disease, did not find any improvement of SBP or HbA1c levels. Participants in this study were also close to target values at baseline, potentially limiting the room for improvement (30).

There are few RCTs that have incorporated text messages as part of a primary prevention strategy targeting multiple CVD risk factors. Several studies have found a decrease in both systolic and diastolic blood pressure, but data are limited when it comes to other risk factors (13, 15). A recent systematic review and meta-analysis found that text messaging interventions had a positive impact on cardiovascular risk factor control, since 61% of the selected studies demonstrated improvements in various risk factors (15). A few other studies have found modest improvements in BMI, weight, waist circumference and physical activity as well as a small decrease in LDL cholesterol and HbA1c (13, 16, 26, 31–33). However, the included studies reported mixed results, likely due to small and heterogeneous study populations, diverse interventions, and their limited individual impact (15). To conclude, there is evidence of text messaging improving specific health behaviors and certain CVD risk factors, but its overall effect on broader risk factor control including HbA1c, weight management, and lipid profile, may be limited. Our results are in line with the existing evidence, adding data from a Swedish context.

Self-rated health is a widely used and valid measure of a person’s overall perception of their health, usually by rating on a scale. It is well established that psychosocial factors influence cardiovascular health and that poor SRH is associated with increased risk of CVD and all-cause mortality (34). Poor SRH is also independently associated with increased risk of CVD and mortality among individuals with hypertension (35). Whether inclusion of SRH into existing CVD risk prediction tools adds predictive value remains unknown. To our knowledge, the effect of health-promoting text messages in CVD risk improvement has not been previously investigated among patients with poor SRH or sedentary lifestyle. Our results provide new insights and are consistent with previously reported results that found an effect on lowering diastolic blood pressure in these subgroups. This is noteworthy since these subgroups might be difficult to reach with usual preventive strategies (14).

It is well known that physical activity reduces insulin resistance and HbA1c levels among patients with T2DM (36). A systematic review and meta-analysis found that structured exercise achieved −0.67% (≈ − 7 mmol/L) reduction in HbA1c; while advice on physical activity had small or no effect unless combined with dietary advice (37). Furthermore, the effect of lifestyle-focused text messaging to improve glycemic control among patients with T2DM was assessed in a meta-analysis, concluding that the intervention significantly improved HbA1c, with an overall reduction of −0.38% (≈ − 4 mmol/L) (31). Among individuals with diabetes, a 0.5% or greater reduction in HbA1c has been linked to a lower risk of cardiovascular events (38). Although HbA1c reductions in individuals without diabetes tend to be smaller than those seen in people with T2DM, evidence suggests that physical activity interventions can still be associated with modest improvements in HbA1c (39, 40). These findings support the effect of physical activity on glycemic control outside manifest diabetes, while highlighting the rather small effect size. This is also reflected in our results, with normal HbA1c levels in the overall study population at baseline and only a slight reduction in the intervention group that might be associated with increased physical activity.

This study is the first Scandinavian RCT investigating the effects of health-promoting text messages among patients with hypertension in primary care. The study design, being a multicenter RCT with a low dropout rate, is a considerable strength. Feasibility was established through a pilot study conducted prior to the PUSHME trial (19). We tried to include patients from diverse socioeconomic segments and from both urban and rural areas by recruiting participants from primary health care centers in different regions. The text messages were designed according to motivational interviewing and in collaboration with experts on lifestyle advice and further improved after feedback from participants in the pilot trial. Moreover, the messages were sent at a reasonable frequency as per previous research with the aim of increasing knowledge and aiding patient empowerment (41, 42). A key limitation of the present study is that the original power calculation was based on the primary outcome (blood pressure) rather than the secondary outcomes reported in this study, and the analyses were therefore exploratory. Secondary outcomes were analyzed without adjustment for multiple comparisons (e.g., Bonferroni) to allow detection of potentially meaningful trends across metabolic risk factors, as such rigorous correction may increase the risk of type II error in this context. However, the absence of adjustment also increases the risk of type I error. Accordingly, the observed HbA1c reduction should be interpreted cautiously, considered hypothesis-generating and not overemphasized in terms of clinical impact. The drop-out rate was somewhat imbalanced, 23 participants in the control group compared to five in the intervention group, with the risk of introducing bias, especially affecting the composite CVD risk measurement that contained several imputed variables. Overall, the participants were in relatively good health and did not exhibit significant cardiovascular strain, which reduces the possibility for improvement and raises the question of healthy volunteer bias. It is possible that the difference between groups would have been more substantial in a population with moderate to high cardiovascular risk (10). Our choice of standard care reflects usual clinical practice in Swedish primary care, where patients with stable hypertension typically are offered annual follow-up and medication management but no structural behavioral interventions. However, the participants’ general practitioners (GPs) responsible for their usual care were also informed about the lab results and could have acted on these, for example, prescribing a statin in case of dyslipidemia. Also, information on other healthcare encounters during the study period was not collected. Lifestyle advice from GPs is considered part of standard care; however, the extent to which such advice was provided to the participants remains unknown and we cannot exclude the possibility that participants may have received such counselling according to guidelines during their annual routine check-up. Since the whole study population decreased their metabolic- and CVD risk, the Hawthorne effect or performance bias must also be considered. However, it can also be an indication that assessment and discussion about lifestyle and cardiovascular risk may contribute to increased awareness and lifestyle modification, which emphasizes the importance of primary care preventive work with individuals at risk. This is also supported by previous studies suggesting that educational interventions directed at patients significantly can affect adherence to lifestyle modifications and that support from healthcare professionals increases the effectiveness of mHealth interventions (11, 41, 43). Moreover, there is the potential for contamination bias, as the participants in the intervention group could have shared the text messages with the control group, although it is unlikely that this would have affected the result. Text messages on dietary advice were included, but the study data collection did not comprise a measure of dietary habits. This was because it was challenging to identify an adequate and feasible method for assessing dietary habits in a primary care setting, yet this would have been interesting to investigate. A limitation of the composite CVD risk outcome is the use of predetermined improvement thresholds. While these cut-offs were clinically meaningful at the individual level, they were not validated within a composite risk measurement. As a result, modest but potentially relevant improvements that did not meet these thresholds may not have been captured, possibly leading to an underestimation of the intervention effect.

Future research could investigate which specific lifestyle behaviors contribute most to CVD risk prevention, and through which intermediate risk factors these effects are mediated. It is also important to further explore whether this type of intervention may be particularly effective when targeting certain subgroups, such as individuals with sedentary lifestyles, poor SRH or additional psychosocial risk factors. In addition, the long-term effects of the intervention on clinical outcomes remain unclear, and further research is needed to determine whether it can lead to sustained behavior change. The usability and acceptability of health-promoting text messages have been previously evaluated, showing favorable results (16, 44). The cost-effectiveness of text messaging was evaluated in the TEXT ME study, which concluded that the intervention was cost-effective; however, comprehensive data on cost-effectiveness across different settings and populations remain limited (16, 45).

## 5 Conclusion

Health-promoting text messages did not improve cardiovascular risk in the total primary care study population but showed a small effect among participants with sedentary lifestyle or poor self-rated health. Moreover, the intervention did not show clinically significant results in lowering metabolic risk, although a small statistically significant difference in HbA1c was found between the intervention and control group. Even modest effects on cardiovascular risk factors may have important clinical and public health implications, particularly if they influence multiple risk factors simultaneously or can be delivered broadly at low cost. Digital interventions show promise, but further research is needed to determine which target groups benefit most, which risk factors are affected, and through which lifestyle behaviors the effects are mediated.

## Data Availability

The data are not publicly available due to restrictions imposed by the ethical approval and applicable data protection regulations. Requests for access to non-public study data may be directed to the corresponding author and will be subject to assessment regarding the need for additional ethical approval and data protection review before any data can be shared.
